# Periodontitis in Psoriatic Patients: Epidemiological Insights and Putative Etiopathogenic Links

**DOI:** 10.3390/epidemiologia5030033

**Published:** 2024-07-26

**Authors:** Federica Di Spirito, Maria Pia Di Palo, Antonio Rupe, Federica Piedepalumbo, Alessandra Sessa, Giuseppina De Benedetto, Serena Russo Barone, Maria Contaldo

**Affiliations:** 1 Department of Medicine, Surgery and Dentistry, University of Salerno, Via S. Allende, 84081 Baronissi, SA, Italy; antoniorupe@virgilio.it (A.R.); f.piedepalumbo1@studenti.unisa.it (F.P.); a.sessa102@studenti.unisa.it (A.S.); giusydb15@gmail.com (G.D.B.); s.russo122@studenti.unisa.it (S.R.B.); 2 Multidisciplinary Department of Medical-Surgical and Odontostomatological Specialties, University of Campania “Luigi Vanvitelli”, 80138 Naples, NA, Italy; maria.contaldo@unicampania.it

**Keywords:** inflammation, microbiome, periodontal disease, periodontitis, psoriasis, skin disease

## Abstract

Psoriasis, a systemic autoimmune disorder primarily affecting the skin, manifests through erythematous plaques and scales, impacting approximately 2–3% of the global population. Chronic periodontitis, a prevalent oral disease characterized by the destruction of tooth-supporting tissues, affects roughly 10–15% of adults worldwide. Emerging evidence suggests a bidirectional relationship between psoriasis and chronic periodontitis, supported by epidemiological studies indicating a higher prevalence of periodontitis among individuals with psoriasis and vice versa. Both conditions are chronic inflammatory diseases marked by dysregulated immune responses and altered cytokine profiles, notably involving proinflammatory cytokines such as TNF-α and IL-17. Clinical studies highlight a reciprocal impact of treating one condition on the other, underscoring the necessity of interdisciplinary collaboration between dermatologists and periodontists in managing patients with both conditions. This narrative review provides a comprehensive overview of the relationship between psoriasis and chronic periodontitis, examining epidemiological associations, shared inflammatory pathways, genetic insights, microbial dysbiosis, environmental factors, and clinical implications. The review emphasizes the importance of integrated care approaches and the potential for targeted therapeutic interventions to improve both psoriatic and periodontal patient outcomes, advocating for further research into the molecular and cellular mechanisms underpinning the comorbidity of these diseases.

## 1. Introduction

Psoriasis, a systemic autoimmune disorder primarily affecting the skin, is characterized by erythematous plaques and scales. It affects approximately 2–3% of the global population, translating to around 125 million people worldwide [1].

In contrast, chronic periodontitis, a prevalent oral disease, leads to the destruction of tooth-supporting tissues and impacts roughly 10–15% of adults globally.

Emerging evidence suggests a bidirectional relationship between psoriasis and chronic periodontitis, supported by epidemiological studies indicating a higher prevalence of periodontitis among individuals with psoriasis and vice versa [2,3] Both conditions are chronic inflammatory diseases marked by dysregulated immune responses and altered cytokine profiles, particularly involving proinflammatory cytokines such as Tumor Necrosis Factor-alpha (TNF-α) and Interleukin-17 (IL-17), which contribute to tissue damage and inflammation [4].

Clinical studies have highlighted a potential reciprocal impact of treating one condition on the other, underscoring the necessity of interdisciplinary collaboration between dermatologists and periodontists in managing patients with both psoriasis and chronic periodontitis [5]. Despite the growing recognition of the interplay between these conditions, the underlying mechanisms remain incompletely understood.

This narrative review aims to provide a comprehensive overview of the current understanding of the relationship between psoriasis and chronic periodontitis. It will encompass epidemiological associations, shared inflammatory pathways, and clinical implications, offering insights into the complex interplay between these chronic inflammatory diseases and guiding future research and clinical practice.

## 2. Psoriasis

Psoriasis is a global health concern, affecting approximately 2–3% of the population worldwide. Its prevalence varies among different ethnic groups, being higher in Caucasians and lower in certain Asian and African populations. In the United States, it is estimated that around 7.5 million people are affected by psoriasis. The disease can manifest at any age, but it commonly appears between the ages of 15 and 30, with a second peak occurring between 50 and 60 years [6].

Psoriasis is a chronic, immune-mediated inflammatory disease primarily affecting the skin and joints. It manifests as erythematous, scaly plaques that can cause significant physical discomfort and psychological stress. The disease has a relapsing-remitting course, with periods of exacerbation and remission. Although the exact etiology remains unclear, psoriasis is understood to be multifactorial, involving a combination of genetic, immunological, and environmental factors [7].

Genetic predisposition plays a crucial role in the onset and progression of psoriasis. Genome-wide association studies have identified multiple susceptibility loci involved in immune regulation, cytokine signaling, and skin barrier function. Notable among these is the PSORS1 locus on chromosome 6p21, which has consistently been linked to psoriasis across different populations [8]. Genetic polymorphisms in cytokines such as interleukin-1 (IL-1), interleukin-6 (IL-6), and TNF-α have been associated with an increased risk of developing both psoriasis and related systemic diseases, indicating a shared genetic basis for these conditions [6].

Furthermore, recent studies have highlighted the role of the interleukin-23/T-helper-17/IL-17 (IL-23/Th17/IL-17) axis in the pathogenesis of psoriasis. Genetic variations affecting this pathway contribute to the inflammatory processes seen in psoriasis and have implications for targeted therapies. A Mendelian randomization study provided evidence that genetic predisposition to elevated levels of inflammatory cytokines, such as IL-17, is linked to both psoriasis and systemic inflammation, supporting the need for integrated therapeutic approaches [9].

Environmental triggers, including infections, trauma, and stress, can precipitate or exacerbate the condition. Additionally, lifestyle factors such as smoking and obesity are known to influence the onset and severity of psoriasis [10].

The pathogenesis of psoriasis involves an intricate interplay between the immune system and skin cells. Key players in the immune response include T-helper cells, particularly Th1 and Th17 subsets, and dendritic cells. Cytokines such as TNF-α, IL-17, IL-23, and interleukin-22 (IL-22) are pivotal in driving the inflammatory process and the hyperproliferation of keratinocytes. This results in the characteristic thickening and scaling of the skin seen in psoriatic lesions [7]. The disease is initiated by the activation of dendritic cells, which present antigens to T-cells, leading to their differentiation and proliferation. Activated T-cells release a cascade of pro-inflammatory cytokines, perpetuating the inflammatory cycle and recruiting additional immune cells to the skin. This chronic inflammation disrupts normal skin cell turnover, leading to the formation of psoriatic plaques [11].

Psoriasis manifests through a variety of clinical features, primarily characterized by erythematous, scaly plaques. The distribution and severity of lesions can vary significantly among patients, reflecting the heterogeneity of the disease.

Plaque psoriasis is the most common form, accounting for about 90% of cases. It typically presents as well-demarcated, red plaques with silvery-white scales, commonly found on the scalp, elbows, knees, and lower back. These plaques can vary in size and thickness, and scaling is often a prominent feature [12].

Guttate psoriasis is characterized by small, drop-shaped lesions. It often appears suddenly and is frequently associated with streptococcal infections, particularly in children and young adults. These lesions are usually widespread and can cover large areas of the body [10].

Inverse psoriasis occurs in skin folds such as the axillae, groin, and under the breasts. The lesions in inverse psoriasis are smooth, red, and shiny, often lacking the typical scaling due to the moist environment of these areas. This form of psoriasis can be particularly uncomfortable due to the friction and sweating that occurs in these regions [12].

Pustular psoriasis is marked by white pustules surrounded by red skin. It can be localized, primarily affecting the hands and feet, or generalized, which is a more severe form that requires immediate medical attention. The pustules are sterile and can cause significant discomfort [7].

Erythrodermic psoriasis is a rare and severe form of the disease, involving widespread inflammation and exfoliation of the skin over a large area of the body. This form of psoriasis can be life-threatening and often requires hospitalization due to complications such as severe dehydration and hypothermia [12].

Psoriatic nail disease affects up to 50% of patients with psoriasis. Symptoms include pitting, onycholysis (separation of the nail from the nail bed), and subungual hyperkeratosis (thickening under the nail). Nail involvement can be a significant source of discomfort and functional impairment for patients [12].

Approximately 30% of individuals with psoriasis develop psoriatic arthritis (PsA), a chronic inflammatory arthritis that can lead to joint damage and disability if not treated effectively. PsA typically affects the joints of the hands, feet, knees, and spine, causing pain, swelling, and stiffness. The presence of PsA can significantly impact a patient’s quality of life and requires a comprehensive management approach [7].

The severity of psoriasis is often assessed using the Psoriasis Area and Severity Index (PASI) and Body Surface Area (BSA) tools.

The PASI score is a tool used to measure the severity and extent of psoriasis. It takes into account the area of skin involvement and the severity of lesions (erythema, induration, and scaling), and combines these factors into a single score ranging from 0 to 72. Higher scores indicate more severe disease [7].

BSA is another measure used to assess the extent of psoriasis. It estimates the percentage of the body’s surface area affected by psoriasis. For example, one hand (including the palm, fingers, and thumb) represents approximately 1% of BSA. BSA is often used in conjunction with PASI to determine disease severity and guide treatment decisions [7].

### Psoriasis, General Health, and Systemic Diseases

Psoriasis is increasingly recognized as a systemic disease associated with a range of comorbidities that can significantly impact overall health and quality of life [13,14].

Patients with psoriasis have an increased risk of cardiovascular diseases, including myocardial infarction, stroke, and hypertension. Chronic systemic inflammation is believed to contribute to the development of atherosclerosis, leading to cardiovascular complications [6,15]. Psoriasis is also associated with a higher prevalence of metabolic syndrome, characterized by obesity, insulin resistance, hypertension, and dyslipidemia. These conditions collectively increase the risk of cardiovascular disease and diabetes [8].

The chronic inflammatory state in psoriasis can lead to insulin resistance, increasing the risk of developing type 2 diabetes. Studies have shown a higher prevalence of diabetes in patients with moderate to severe psoriasis compared to the general population [7].

Obesity is a significant risk factor for both the development and the severity of psoriasis. Adipose tissue produces pro-inflammatory cytokines that can exacerbate psoriasis and contribute to systemic inflammation [10].

Psoriasis can have profound psychological effects, including depression, anxiety, and social isolation. The visibility of skin lesions can lead to stigmatization and decreased quality of life. Psychological stress, in turn, can exacerbate psoriasis, creating a vicious cycle [6].

Patients with psoriasis are more likely to experience chronic kidney disease and non-alcoholic fatty liver disease (NAFLD), and are at increased risk for certain cancers, particularly lymphomas [8].

Other comorbidities associated with psoriasis include inflammatory bowel disease (IBD) such as Crohn’s disease and ulcerative colitis [8]. Indeed, it is not uncommon for an immune-mediated inflammatory pathology and another inflammatory and/or autoimmune systemic disease to coexist in the same patient, as they result in or are triggered by a loss of regulation of the innate and adaptive functions of the immune system [16], although their etiology is still unclear and is partly related to environmental factors such as infections, trauma and genetic susceptibility [17].

## 3. Oral Status in Psoriatic Patients

### 3.1. Oral Psoriasis

Psoriasis appears to be related to an increase in the prevalence of oral lesions [13]. In fact, patients affected by psoriasis show an increase in the occurrence of abnormal mucosae (74%) compared to healthy individuals (46%) [18]. Certain types of psoriasis, particularly generalized pustular psoriasis and erythrodermic psoriasis, are more frequently associated with oral mucosal lesions [19].

It is often challenging to determine whether an oral lesion in a patient with psoriasis is related to the disease or if it is coincidental. There is no consensus regarding clinical or histopathological criteria for diagnosis, and lesions may vary significantly in appearance among different patients [18]. Accordingly, mucosal lesions in the oral cavity of psoriatic patients can be divided into psoriasis-specific lesions and nonspecific lesions.

Psoriasis-specific oral lesions may precede the development of skin manifestations but usually occur concurrently. Clinically, these lesions can appear as ring-shaped lesions, diffuse erythema, gray or white plaques, or simple edema. These lesions can occur on various oral mucosal surfaces, including the buccal mucosa, tongue, palate, and gingiva [18,20].

Histopathologically, oral psoriatic lesions resemble cutaneous psoriatic plaques. They are characterized by parakeratosis, acanthosis, elongation of rete ridges, and a mixed inflammatory infiltrate. However, these histological features alone are not pathognomonic; they may show organized neutrophilic microabscesses, acute inflammatory infiltrates of the epithelium, or mixed infiltrates composed of neutrophils and lymphocytes in the lamina propria [19,21,22].

Although oral psoriasis is considered uncommon, it can significantly affect patients who experience it. Diagnosing oral psoriasis can be challenging as its clinical appearance often mimics other conditions such as geographic tongue, lichen planus, and candidiasis. Therefore, a thorough clinical history and correlation with cutaneous findings are essential for an accurate diagnosis.

Nonspecific lesions in psoriatic patients are those that can also be found in healthy individuals. The most frequent oral lesion found in psoriatic patients is fissured tongue, followed by geographic tongue. Geographic tongue is characterized by erythematous patches with white, serpiginous borders that can change location, size, and shape over time, often referred to as “migratory glossitis” [21]; erythematous plaques can also appear on the buccal mucosa, lips, and palate, which, though often asymptomatic, can cause a burning sensation or pain, especially when consuming spicy or acidic foods [20]. Fissured tongue, characterized by deep grooves or fissures on the dorsal surface of the tongue, is frequently associated with geographic tongue and can be seen in psoriatic patients [20].

In some cases, psoriatic patients may present with desquamative gingivitis, exhibiting erythematous and desquamative gingival lesions that are painful and prone to secondary infections [20].

Other oral manifestations in this category include leukokeratosis, cheilitis, depapillation of the tongue, fibromas, and erythematous macules [18].

### 3.2. Perceived Oral Health in Psoriatic Subjects

Perceived oral health refers to an individual’s subjective assessment of their oral health status and its impact on daily activities and quality of life. The Oral Health Impact Profile (OHIP) is a widely used tool that provides a comprehensive measure of the social impact of oral health conditions [23].

Studies using the OHIP have generally found that psoriatic patients with oral lesions report higher OHIP scores, indicating a greater negative impact on their oral health-related quality of life. These findings highlight the significant burden that oral psoriasis can place on patients, affecting their daily lives and overall well-being [23].

Pain and discomfort are common complaints among psoriatic patients with oral lesions, particularly when eating, drinking, or speaking. This discomfort can lead to a reduced quality of life and avoidance of certain foods, impacting nutritional intake [24].

Functional impairments caused by oral lesions, such as difficulty in chewing, swallowing, and speaking, can affect daily activities and overall well-being [24].

Additionally, the psychological burden of managing a chronic condition with visible symptoms can contribute to psychological stress, anxiety, and depression, further exacerbating perceived oral health problems [24].

Aesthetic concerns also play a significant role in perceived oral health. Visible oral lesions can lead to self-consciousness, social embarrassment, and reluctance to engage in social interactions [24].

## 4. Periodontitis

Periodontitis is one of the most prevalent oral diseases, significantly affecting the adult population worldwide. The prevalence of periodontitis increases with age, and it is estimated that over 50% of adults aged 30 years and older in the United States have some form of periodontal disease. The severe form of periodontitis affects approximately 10–15% of the global population, underscoring its status as a major public health concern due to its association with tooth loss and other systemic conditions [6].

Periodontitis is a chronic inflammatory disease that progressively affects the supporting structures of the teeth, leading to a loss of attachment, bone loss, and ultimately, if left untreated, tooth loss. It is characterized by the destruction of the periodontal ligament and alveolar bone, resulting in the formation of periodontal pockets around the teeth due to the detachment of the gum from the tooth surface.

This condition arises from complex interactions between pathogenic bacteria and the host’s immune response [25]. The primary etiological factor in periodontitis is the accumulation of periodontal biofilm, which harbors a variety of pathogenic bacteria. These bacteria trigger an immune response that, while intended to protect the body, results in the destruction of periodontal tissues. Key bacterial species involved in periodontitis include *Porphyromonas gingivalis*, *Tannerella forsythia*, and *Aggregatibacter actinomycetemcomitans*. These pathogens disrupt the host–microbe homeostasis, leading to a chronic inflammatory state.

The pathogenesis of periodontitis involves both bacterial factors and host factors. Bacterial components such as lipopolysaccharides stimulate the production of inflammatory mediators like IL-1, IL-6, and TNF-α, which in turn activate immune cells. These immune cells release enzymes and reactive oxygen species that degrade the extracellular matrix and bone, resulting in tissue destruction and disease progression.

Genetic predisposition plays a significant role in the development and progression of periodontitis, considering that up to 50% of the variance in periodontitis susceptibility can be attributed to genetic factors [17]. Several genetic polymorphisms have been associated with an increased risk of periodontitis, particularly those involved in immune response and inflammation. In detail, key genetic markers and pathways include variations in the IL-1 gene cluster, which have been linked to an increased inflammatory response, leading to greater tissue destruction in periodontitis patients [26,27]. Additionally, genetic variations in the TNF-α gene are associated with increased susceptibility to periodontitis and more severe disease progression [28,29]. Polymorphisms in matrix metalloproteinases (MMPs), enzymes that degrade extracellular matrix components, can influence their expression and activity, contributing to variability in disease severity among individuals [30,31,32]. Furthermore, the vitamin D receptor (VDR) gene plays a role in immune response and bone metabolism, with polymorphisms in the VDR gene linked to an increased risk of periodontitis, suggesting a role of vitamin D metabolism in periodontal health [33,34].

Clinically, periodontitis presents with several distinct signs and symptoms. Chronic periodontitis is the most prevalent form, typically seen in adults. It progresses slowly and is often associated with the accumulation of periodontal biofilm and calculus. Patients often exhibit gingival inflammation, characterized by red, swollen gums that may bleed during brushing or flossing, periodontal pockets, and halitosis; as the disease progresses, tooth mobility can occur due to the loss of supporting bone structure, and in advanced cases, tooth loss becomes inevitable [25,35]. Necrotizing periodontal disease, including both gingivitis and periodontitis, is characterized by rapid onset of pain, gingival ulceration, and necrosis. It is often associated with systemic conditions such as Human Immunodeficiency Virus (HIV), malnutrition, and immunosuppression. Clinical features include the presence of ulcerated and necrotic papillae, gingival bleeding, and a fetid odor [36,37].

Once diagnosed as per currently accepted criteria, periodontitis is staged and graded to assess the severity and progression of the disease, aiding in treatment planning. Staging is based on the extent of periodontal destruction, with stage I (initial periodontitis) representing early stages with mild attachment loss, stage II (moderate periodontitis) involving moderate attachment loss with some bone loss, stage III (severe periodontitis with potential for tooth loss) characterized by severe attachment loss and significant bone loss, potentially affecting tooth stability, and, finally, stage IV (advanced periodontitis with extensive tooth loss and potential for loss of dentition) involving extensive attachment and bone loss, affecting multiple teeth [38]. Grading, meanwhile, reflects the rate of disease progression and risk factors, with grade A (slow progression) showing minimal evidence of disease progression over five years, grade B (moderate progression) indicating evidence of disease progression over five years, often with the presence of risk factors such as smoking or diabetes, and grade C (rapid progression) characterized by rapid disease progression with significant clinical attachment loss and bone destruction [38].

### Periodontitis, General Health, and Systemic Diseases

Periodontitis has been associated with various systemic conditions, emphasizing the importance of periodontal health in overall well-being [39]. The bidirectional relationship between periodontitis and systemic diseases is mediated by chronic inflammation and the systemic spread of periodontal pathogens [17,40].

Periodontitis induces a chronic inflammatory response in the periodontal tissues, characterized by the release of pro-inflammatory cytokines such as interleukin-1 beta (IL-1β), TNF-α, and IL-6. These cytokines enter the systemic circulation, leading to a state of low-grade chronic inflammation that can exacerbate or contribute to the pathogenesis of various systemic conditions. For example, elevated levels of C-reactive protein (CRP), a marker of systemic inflammation, are frequently observed in individuals with periodontitis and are associated with an increased risk of cardiovascular diseases [41].

Periodontal pathogens such as *Porphyromonas gingivalis*, *Tannerella forsythia*, *Fusobacterium nucleatum*, and *Treponema denticola* can enter the bloodstream through ulcerated periodontal pockets. Once in the systemic circulation, these pathogens and their virulence factors can invade distant organs, contributing to the development or exacerbation of systemic diseases. For instance, *P. gingivalis* has been detected in atherosclerotic plaques, suggesting a direct microbial contribution to the development of cardiovascular diseases [17].

The association between periodontitis and cardiovascular diseases, including atherosclerosis, myocardial infarction, and stroke, is well-documented. The chronic inflammatory state induced by periodontitis promotes endothelial dysfunction, a precursor to atherosclerosis. Moreover, periodontal pathogens can directly invade endothelial cells, promoting the formation of atheromatous plaques. This dual mechanism underscores the significant impact of periodontal health on cardiovascular risk [40].

There is a well-established bidirectional relationship between periodontitis and diabetes. Diabetic patients are more susceptible to periodontitis, and the presence of periodontal disease can exacerbate glycemic control, leading to a vicious cycle of worsening oral and systemic health [8]. Hyperglycemia in diabetic patients enhances the inflammatory response to periodontal infection, leading to more severe periodontal destruction. Conversely, the systemic inflammation associated with periodontitis exacerbates insulin resistance and glycemic control, creating a vicious cycle that complicates the management of diabetes [42].

Chronic respiratory conditions, such as chronic obstructive pulmonary disease (COPD) and pneumonia, are also linked to periodontitis. The aspiration of periodontal pathogens into the lungs can lead to infections and inflammatory responses in the respiratory tract. Studies have shown that improving periodontal health can reduce the incidence and severity of respiratory infections, highlighting the importance of oral hygiene in respiratory disease management [43].

Pregnant women with periodontitis have a higher risk of adverse pregnancy outcomes, including preterm birth, low birth weight, and preeclampsia. The systemic inflammation and bacterial infection associated with periodontitis are thought to contribute to these complications [44]. The systemic inflammatory response and potential hematogenous spread of periodontal pathogens to the placental tissues are thought to contribute to these complications. Periodontal treatment during pregnancy has been shown to reduce the risk of these adverse outcomes, underscoring the importance of periodontal care in prenatal health [44].

Emerging evidence suggests that periodontitis may also be linked to cognitive decline and Alzheimer’s disease. Chronic inflammation and the systemic spread of periodontal pathogens to the brain may contribute to the development of neurodegenerative conditions. Studies have identified periodontal pathogens in the brain tissues of Alzheimer’s patients, supporting the hypothesis that periodontitis could play a role in cognitive impairment [45].

The concept of the oral–systemic health axis highlights the interconnected nature of oral health and overall health. Maintaining periodontal health is not only crucial for preserving teeth but also for preventing and managing systemic diseases. The bidirectional relationship between periodontitis and systemic diseases underscores the need for an integrated approach to healthcare, where dental professionals and medical practitioners collaborate to address the interrelated aspects of patient health [46]

Additionally, some studies suggest that periodontal disease may be related to oral squamous cell carcinoma and colorectal cancer, as well as other immune-mediated inflammatory diseases [47]. However, apart from a common imbalance of the inflammatory cytokine network between periodontitis and immune-mediated inflammation, their possible link remains controversial [17].

## 5. Periodontitis in Psoriatic Patients

### 5.1. Epidemiological Insights

Patients suffering from both periodontitis and psoriasis generally exhibited poorer oral health scores, indicating the potential impact of periodontitis on the quality of life of psoriasis patients [47]. In detail, individuals with psoriasis exhibited a significantly higher prevalence of periodontitis compared to their non-psoriatic counterparts, with reported odds ratios ranging from 1.4 to 4.1 in various studies [10,48]. Additional research has found that psoriasis patients had a markedly higher prevalence of periodontitis compared to controls [44]. Accordingly, a study involving 69 psoriasis patients and 74 healthy controls found that periodontitis was significantly more common among those with psoriasis, with severe periodontitis present in 87.1% of psoriasis patients compared to 58.1% of controls [49]. Similarly, another study by Ganzetti et al. [50] reported that out of 82 psoriasis patients, 72 had periodontitis, compared to 54 out of 89 in the control group, demonstrating a significantly higher prevalence of periodontitis in psoriatic individuals [51]. Sharma et al. (2014) [52] also observed a significant prevalence of periodontitis among psoriatic patients. In their study, 23 out of 60 psoriasis patients had periodontitis, whereas only 9 out of 45 controls were affected [52].

As a counterpart, a population-based study in the United States demonstrated a greater prevalence of psoriasis among individuals with periodontitis compared to those without this oral condition [53]. Additionally, patients diagnosed with chronic periodontitis appeared more likely to have psoriasis compared to those without periodontal disease [4,54]. Moreover, a meta-analysis conducted by Zhang et al. (2022) [51] demonstrated that individuals with psoriasis had a 1.72-times higher chance of having periodontitis than controls in univariate analysis, although the association was not significant in multivariate analysis.

A case-control study conducted by Costa et al. demonstrated that psoriatic patients had a 1.4-times higher likelihood of developing periodontal disease, as confirmed by common periodontal indexes such as Bleeding on Probing (BOP), Periodontal Probing Depth (PD), and Clinical Attachment Loss (CAL) [47]. Similarly, a nationwide cohort study in Denmark identified a significantly elevated risk of periodontitis among individuals with psoriasis, even after adjusting for confounders such as age, gender, socioeconomic status, and smoking habits [10]. These findings were confirmed by a recent meta-analysis reporting that psoriasis patients showed a more than twofold increase in the odds of developing periodontal disease, with nearly one-third of these patients suffering from periodontitis of varying severity [47].

The risk of developing psoriasis has been demonstrated to be six times higher in psoriatic patients who smoke, compared to non-smoking psoriasis patients [17]. As conceivable, the risk of developing periodontitis has been demonstrated to be six times higher in psoriatic patients who smoke, compared to non-smoking psoriasis patients [17,55].

Moreover, not only the incidence and prevalence but also the severity of periodontitis has been correlated to that of psoriasis [17], as also supported by data from a case-control study highlighting that individuals with severe psoriasis are more likely to experience advanced periodontitis, suggesting a correlation between disease severity and periodontal health [44]. In addition, Ganzetti et al. [50] highlighted that 24% of psoriasis patients had moderate to severe periodontitis, compared to only 13% of the control group. Further supporting these findings, a study conducted in Norway found that psoriasis patients had a significantly higher prevalence of moderate and severe periodontitis (24%) compared to healthy controls (10%) [56]. Additionally, 36% of psoriasis cases had one or more sites with radiographic bone loss of ≥3 mm, compared to 13% of controls [51]. Psoriasis patients had significantly more sites with clinical attachment loss (CAL) of ≥3 mm and ≥5 mm, fewer teeth, and a higher Decayed, Missing, and Filled Teeth (DMFT) index compared to controls. Severe periodontitis was more frequent among psoriasis patients (87.1%) compared to controls (58.1%), and severe periodontitis was identified as a risk indicator for psoriasis after adjusting for sex, age, race, and smoking habits (odds ratio: 3.7, 95% confidence interval: 1.5–9.0, *p* < 0.003) [49].

### 5.2. Putative Etiopathogenic Links

The link between psoriasis and chronic periodontitis stems from a multifaceted interplay of genetic, immunological, microbial, and environmental factors, underscoring a complex etiology that fosters their bidirectional relationship [6,8].

#### 5.2.1. Periodontitis in Psoriatic Patients: Genetic Factors

Numerous susceptibility genes have been identified in periodontitis, though none appear to overlap with those found in psoriatic patients. The gene most frequently implicated in the development and progression of periodontitis in Caucasians is the one encoding IL-1. This indicates that, despite the lack of definitive genetic links between psoriasis and periodontitis, both diseases exhibit mutations in genes responsible for cytokine production, thereby partially explaining the immune dysregulation observed in both conditions on a genetic basis [57].

As a counterpart, research has sought to identify shared chromosomal regions in patients with psoriasis, focusing on genes containing psoriasis-susceptibility (PSORS) loci. According to multiple studies, psoriasis is primarily attributable to genetic variations in PSORS1, located on chromosome 6 within the Major Histocompatibility Complex (MHC). Specifically, the HLA-C gene is most significantly affected. Additionally, recent findings implicate a gene in PSORS2 on chromosome 17q in the pathogenesis of psoriasis. Notably, studies have identified a mutation in the Caspase Recruitment Domain-Containing Protein 14 (CARD14) gene within this region [57].

Based on this evidence, genetic predisposition holds significance in the onset and progression of both conditions. Notably, genome-wide association studies have pinpointed common genetic variants implicated in immune regulation, cytokine signaling, and tissue remodeling, shedding light on shared susceptibility mechanisms [6,8]. Such genetic predispositions likely contribute to the dysregulated immune responses and altered inflammatory pathways observed in both psoriasis and chronic periodontitis.

Moreover, recent studies have identified specific genetic polymorphisms, including those related to IL-1, IL-6, and TNF-α, that are associated with an increased risk for both diseases. These polymorphisms contribute to a heightened inflammatory response and a greater susceptibility to both conditions [6,44]. Additionally, genetic factors influencing the antigen presentation, innate immunity, T-cell development, and IL-23/Th17/IL-17 axis, which is pivotal in both diseases, further illustrate the genetic link between psoriasis and periodontitis [44,57].

Furthermore, a recent Mendelian randomization study has provided evidence suggesting that a genetic predisposition to inflammatory pathways, such as those involving TNF-α and IL-17, may underlie the observed comorbidity between periodontitis and psoriasis. This study highlights that shared genetic factors contribute significantly to the pathogenesis of both conditions, supporting the need for integrated therapeutic approaches targeting these common pathways [9].

#### 5.2.2. Periodontitis in Psoriatic Patients: Immunological Dysregulation

Immunological dysregulation is a hallmark of both psoriasis and periodontitis, characterized by an imbalance between proinflammatory and anti-inflammatory cytokines.

The connection between psoriasis and chronic periodontitis involves shared genetic, immunological, microbial, and environmental factors. Dysregulated immune responses to microbiota at epithelial surfaces and shared genetic predispositions affecting dendritic cells and Toll-like receptor (TLR) expression are proposed mechanisms [17,47,48]. Polymorphisms in genes related to the IL-23/Th17 pathway may predispose individuals to both conditions by enhancing pro-inflammatory responses [44].

These dysregulated responses exacerbate tissue damage and inflammation, perpetuating disease progression. Inflammatory events in the periodontium influence systemic immune responses, enhancing the development of diseases such as psoriasis [47]. Both conditions share similar cellular signaling pathways, including the activation of dendritic cells and the production of pro-inflammatory cytokines like TNF-α and IL-17, driving chronic inflammation and bone resorption [17,48].

Elevated levels of IL-17 and other pro-inflammatory cytokines in the saliva of psoriasis patients further support this link [13]. Psoriasis involves abnormal cytokine proliferation and secretion from keratinocytes, with IL-1β playing a significant role in systemic immune responses. The presence of IL-1β in psoriatic keratinocytes creates a positive feedback loop, leading to higher production of mature IL-1β, while periodontal immune homeostasis involves commensal microorganisms and bacterial environment exposure, stimulating inactive IL-1β precursors in healthy tissue macrophages. These findings suggest psoriatic immune responses may include IL-1β signals, supporting the association between psoriasis and periodontitis [14].

Accordingly, both diseases involve dendritic-cell-mediated T-cell activation, leading to increased production of proinflammatory cytokines such as TNF-α, IL-1β, IL-17, IL-22, and interferon-γ (IFN- γ) [17,48]. This overproduction fosters chronic inflammation and tissue degradation in both conditions [4,54,58]. Recent studies have highlighted that both diseases also show elevated levels of IL-23, which is critical for maintaining Th17 cells and promoting IL-17 production [47].

Indeed, in psoriasis and periodontitis, immune activation is driven by macrophages, Th17 cells, and dendritic cells, with secondary involvement of adaptive immunity [17,48]. Consistently, both conditions exhibit a predominantly neutrophilic immune response, with increased neutrophilic infiltration seen in psoriatic plaques and gingival lesions [47], and IL-17, TNF-α, and interleukin-36 (IL-36) amplify inflammatory pathways and promote neutrophil influx, particularly in psoriasis [9]. Furthermore, the presence of IL-17 in both diseases not only drives inflammation but also induces osteoclastogenesis, leading to bone resorption and subsequent tissue damage [47].

Specifically, IL-17 plays a crucial role in the pathogenesis of psoriasis, contributing to both the initiation and perpetuation of the inflammatory processes characteristic of the disease, and specifically driving the inflammatory processes that lead to keratinocyte hyperproliferation, neutrophil recruitment, and chronic inflammation [59]. Its central role in disease mechanisms makes it a valuable target for therapeutic strategies aimed at controlling and mitigating psoriasis [59].

In periodontitis, IL-17 modifies the oral environment by exacerbating inflammation and promoting microbial dysbiosis. The production of IL-17, which depends on IL-23, leads to excessive bacterial growth, while its inhibition has been shown to reduce bacterial proliferation, highlighting IL-17’s role in maintaining pathogenic microbial communities [44].

Table 1 illustrates IL-17’s putative role in psoriasis and periodontitis pathogenesis.

Moreover, the shared increase in TLRs in both diseases suggests these receptors play a fundamental role in their pathogenesis. Toll-Like Receptor 2 (TLR2) and Toll-Like Receptor 4 (TLR4) expression in response to bacterial antigens like *Porphyromonas gingivalis* can amplify inflammatory responses [14].

#### 5.2.3. Periodontitis in Psoriatic Patients: Microbial Dysbiosis

Microbial dysbiosis is a significant shared feature of both psoriasis and chronic periodontitis, with disruptions in microbial composition and diversity playing a crucial role in disease pathogenesis [64].

In psoriasis, dysbiosis of the skin microbiota, particularly the overabundance of pathogenic bacteria like *Staphylococcus aureus*, can trigger inflammatory reactions and exacerbate disease severity [65].

Similarly, imbalances in the oral and periodontal microbiota, characterized by shifts between pathogenic and commensal bacteria, are pivotal in the development and progression of chronic periodontitis [54]. Indeed, it is well known that periodontal pathogens, such as *Porphyromonas gingivalis*, *Tannerella forsythia*, and *Aggregatibacter actinomycetemcomitans*, play critical roles in the onset and progression of periodontitis by disrupting host–microbe homeostasis and triggering sustained inflammatory responses [47]. Although an imbalance between commensal and pathogenic bacteria is essential for disease development, it is the inflammatory response against these bacteria that leads to cell-mediated degradation of periodontal tissue, ultimately resulting in tooth loss [9].

New evidence suggests that dysbiotic periodontal microbiota may contribute to the systemic inflammatory burden seen in psoriasis. For example, specific microbial species in the oral cavity, especially from the periodontal niche, have been linked to increased systemic inflammation in psoriatic patients [44]

In addition, a clear association between gastrointestinal dysbiosis and psoriasis has been reported. Psoriasis also appears to be related to other gastrointestinal diseases, such as ulcerative colitis, Crohn’s disease, and irritable bowel syndrome (IBS). Some studies have suggested that periodontitis may be a consequence of gastrointestinal dysbiosis. This hypothesis stems from the fact that both are due to a lack of balance in the microbiome [14].

Moreover, recent studies have identified specific bacterial species and virulence factors that may play a role in disrupting immune homeostasis, further linking periodontal dysbiosis to psoriatic inflammation [66]. The link between microbial dysbiosis and immunological dysregulation is further supported by the shared immunopathological mechanisms in both diseases.

The IL-23/Th-17/IL-17 axis is a common pathway suggesting that inflammation in distant sites, such as the skin or joints, can be driven by adaptive immune responses to oral pathogens. Accordingly, the presence of oral microbiota DNA in the synovial fluid of patients with psoriatic arthritis supports the hypothesis that periodontal infections can exacerbate systemic inflammatory conditions, including psoriasis [47]. In addition, inflammatory mediators in the periodontium, such as TNF-α, interleukins, and prostaglandins, when released into the bloodstream, can contribute to systemic inflammation [44].

Based on the already described observation that both conditions are characterized by an exaggerated immune response against superficial epithelial microbiota, it has been proposed that periodontal pathogens, such as *Porphyromonas gingivalis* and *Prevotella intermedia*, may trigger and exacerbate skin and joint manifestations of psoriasis by acting as superantigens, thereby perpetuating the inflammatory cycle [47].

**Figure 1:**
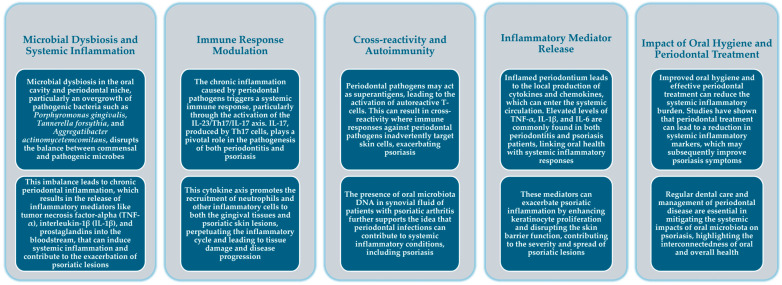
Periodontal microbiota in the onset and progression of psoriasis.

Figure 1 synthesizes the potential role of the periodontal microbiota in the onset and progression of psoriasis.

#### 5.2.4. Periodontitis in Psoriatic Patients: Environmental Factors

Several environmental factors, such as smoking, obesity, and psychological stress, play a significant role in influencing immune responses, thereby exacerbating disease susceptibility and severity in psoriasis and periodontitis [11,67,68]. Therefore, understanding the impact of these environmental factors is crucial in managing and mitigating the progression of psoriasis and periodontitis.

Recent studies emphasize that certain environmental triggers, including infections and medication use—specifically beta-blockers and anti-malarial drugs—can aggravate psoriasis and lead to disease flares [10]. In particular, streptococcal throat infections have been identified as common triggers for psoriasis flares, especially in pediatric cases [10].

Infections are known to play a significant role in the exacerbation of both psoriasis and periodontitis. Streptococcal infections, in particular, are well-documented triggers for guttate psoriasis, highlighting the importance of microbial factors in the pathogenesis of psoriasis [10]. In periodontitis, the role of bacterial infection is central, with periodontal pathogens such as *Porphyromonas gingivalis* contributing to both local tissue destruction and systemic inflammatory responses [40]. The cross-talk between these infections and the host immune system can amplify inflammatory pathways, worsening both conditions.

Medication use also has a notable impact on the course of both diseases. Medications such as beta-blockers and anti-malarial drugs have been shown to exacerbate psoriasis, potentially by altering immune responses and promoting inflammatory pathways [10]. Similarly, certain medications can affect periodontal health, either through direct effects on the gingival tissues or by altering systemic inflammatory responses [40].

Moreover, exposure to certain environmental pollutants, such as particulate matter and heavy metals, has been shown to exacerbate both psoriasis and periodontitis by enhancing inflammatory pathways [44]. Pollutants can trigger oxidative stress and inflammation, leading to exacerbation of skin and periodontal conditions.

Psychological stress is another critical factor that modulates immune responses and exacerbates the severity of both psoriasis and periodontitis through enhanced inflammatory pathways [47]. Chronic stress triggers not only exacerbate inflammation and tissue damage but also create conditions conducive to disease progression. Chronic stress has been associated with increased production of pro-inflammatory cytokines and reduced efficacy of the immune response, thereby worsening the clinical outcomes of both diseases [44]. Stress-induced alterations in immune function can lead to heightened inflammatory responses in both the skin and periodontal tissues.

Obesity is implicated as a contributing factor to the severity of psoriasis and is recognized as a risk factor for periodontitis. The systemic inflammation induced by adipose tissue is believed to be a significant underlying mechanism [10,47]. Additionally, obesity-related metabolic changes, including insulin resistance and dyslipidemia, further contribute to the chronic inflammatory state observed in both conditions [44]. Obesity is associated with elevated levels of pro-inflammatory cytokines, such as TNF-α and IL-6, which play a role in the pathogenesis of both psoriasis and periodontitis.

Recent studies have also emphasized that specific dietary factors, such as high sugar intake and low consumption of fruits and vegetables, may contribute to the inflammation observed in both conditions [44]. Diets high in refined sugars and fats can promote systemic inflammation, while a diet rich in fruits and vegetables can provide antioxidants and anti-inflammatory compounds that may mitigate inflammatory responses.

## 6. Discussion and Clinical Implications

**Figure 2:**
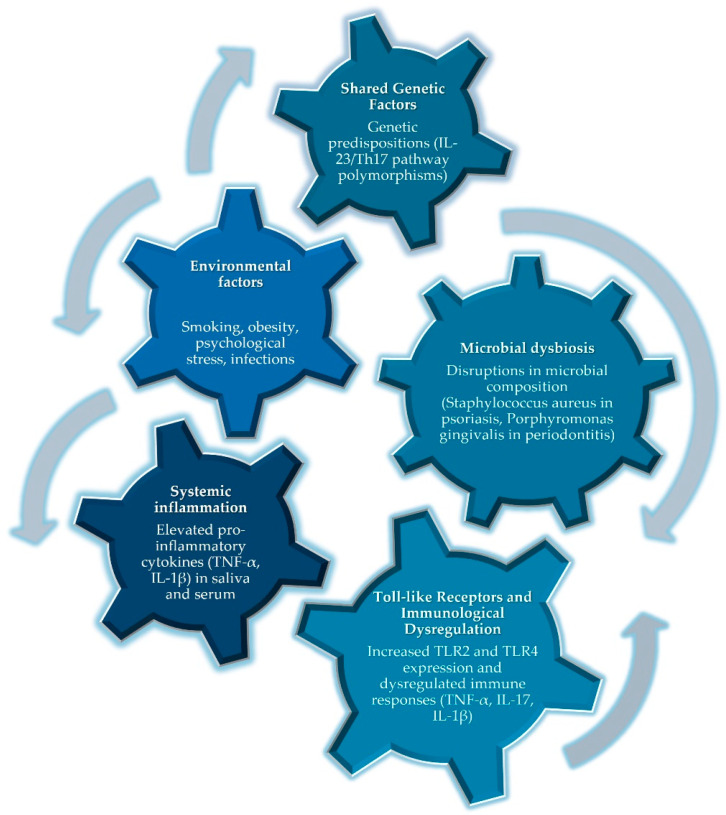
Putative etiopathogenic links contributing to the comorbidity of psoriasis and periodontitis: interconnected pathways and factors.

The relationship between psoriasis and periodontitis seems to be a complex and multifaceted one, marked by shared genetic, immunological, microbial, and environmental factors (Figure 2).

The relationship between psoriasis and periodontitis has been widely discussed in the literature, with several studies suggesting a potential link that can be attributed to common inflammatory pathways and shared risk factors. However, critically evaluating the available evidence reveals significant limitations that weaken the claim of a direct association or causal relationship between these two conditions.

Firstly, many of the studies cited in the literature focus on either psoriasis or periodontitis alone rather than simultaneously examining cohorts with both conditions. This approach undermines the strength of any association claims as it fails to account for specific interaction between psoriasis and periodontitis in the same individuals. For instance, the meta-analysis by Ungprasert et al. [69] found a 1.55-fold increased risk of psoriasis in individuals with periodontitis, but the included studies did not always have cohorts with both diseases [70].

Moreover, the study by Gupta et al. [71] found no statistically significant association between psoriasis and inflammatory periodontal disease when comparing psoriasis patients with healthy controls. Their results suggest that psoriasis patients had better periodontal health than the control group, contradicting the widely accepted notion of a positive association between these diseases [71]. This inconsistency in results emphasizes the need to include only relevant studies in which both diseases occur in the same cohort.

Furthermore, while some studies suggest common pathways, such as immune activation by dysbiotic pathogens and common inflammatory mediators such as IL-33, this does not necessarily prove an associational link. For example, the study by Christophers et al. [70] discusses how immune activation in both diseases involves similar inflammatory pathways. Still, it does not conclusively prove a direct link between periodontitis and psoriasis. The presence of common risk factors such as smoking and obesity adds another layer of complexity, as these factors independently contribute to the manifestation and severity of both diseases without necessarily linking them causally.

Accordingly, the claims of direct association and causation between psoriasis and periodontitis are challenging to verify based on the current evidence. The results of these reviews emphasize the importance of rigorous study designs that include cohorts with both psoriasis and periodontitis to draw more definitive conclusions.

However, understanding the intricate relationship between psoriasis and periodontitis has several critical clinical implications, particularly in the realms of diagnosis, treatment, and interdisciplinary care, requiring a holistic approach to patient care.

It is essential to integrate dental and dermatological care to monitor and manage the inflammatory burden effectively. An integrated care approach involves collaboration between healthcare providers across different specialties. Dermatologists, dentists, and general practitioners should work together to develop a comprehensive treatment plan. This approach ensures that both conditions are addressed simultaneously, which can lead to better overall health outcomes.

Genetic predisposition plays a critical role in both psoriasis and periodontitis, with genome-wide association studies pinpointing common genetic variants involved in immune regulation, cytokine signaling, and tissue remodeling. Immunological dysregulation is a hallmark of both psoriasis and periodontitis. Both diseases involve dendritic-cell-mediated T-cell activation, leading to increased production of proinflammatory cytokines such as TNF-α, IL-1β, IL-17, IL-22, and IFN-γ. This cytokine overproduction fosters chronic inflammation and tissue degradation in both conditions [4,17,48,54,58]. Elevated levels of IL-23, critical for maintaining Th17 cells and promoting IL-17 production, are also seen in both diseases [47]. The presence of IL-17 drives inflammation and induces osteoclastogenesis, leading to bone resorption and tissue damage in periodontitis, while also driving keratinocyte hyperproliferation, neutrophil recruitment, and chronic inflammation in psoriasis [59].

Advances in understanding the shared molecular pathways in psoriasis and periodontitis pave the way for targeted therapeutic interventions. Treatments targeting the IL-23/Th17/IL-17 axis, which plays a pivotal role in both diseases, may offer effective solutions. Biologics that inhibit these pathways can potentially control inflammation more efficiently in patients suffering from both conditions. These targeted therapies can lead to better management of symptoms and a reduction in disease severity [59].

Microbial dysbiosis is another characteristic shared between psoriasis and chronic periodontitis, with disruptions in microbial composition and diversity playing a crucial role in disease pathogenesis [72]. In psoriasis, skin microbiota dysbiosis, particularly the overabundance of pathogenic bacteria like Staphylococcus aureus, can trigger inflammatory reactions and exacerbate disease severity [65]. Similarly, imbalances in the oral and periodontal microbiota, characterized by shifts between pathogenic and commensal bacteria, are pivotal in the development and progression of chronic periodontitis [47,54]. The presence of specific microbial species in the oral cavity and the periodontal niche has been linked to increased systemic inflammation in psoriatic patients [44].

Regular dental check-ups and periodontal treatments should be a routine part of the healthcare regimen for patients with psoriasis, and vice versa [73]. Regular screening for periodontal disease in psoriatic patients and vice versa can help in early detection and management, thus preventing complications. Periodontal treatment can reduce systemic inflammation, potentially alleviating the severity of psoriasis [4,47].

Environmental factors, such as smoking, obesity, and psychological stress, significantly influence immune responses, exacerbating disease susceptibility and severity in psoriasis and periodontitis [11,67,68]. Infections, particularly streptococcal throat infections, are well-documented triggers for guttate psoriasis and play a role in exacerbating periodontitis by amplifying inflammatory pathways [10,40]. Medication use, such as beta-blockers and anti-malarial drugs, can also aggravate psoriasis and impact periodontal health by altering immune responses and promoting inflammatory pathways [10,40]. Psychological stress exacerbates both conditions through enhanced inflammatory pathways, increasing the production of pro-inflammatory cytokines and reducing immune response efficacy [44,47]. Obesity is another significant factor, contributing to the severity of both diseases through systemic inflammation induced by adipose tissue and metabolic changes such as insulin resistance and dyslipidemia [10,44,47].

Given the significant role of environmental factors in the exacerbation of both diseases, lifestyle modifications can play a crucial part in disease management [74]. Smoking cessation programs, stress management techniques, and weight management strategies should be integral components of the treatment plan. Educating patients about the impact of diet, such as reducing sugar intake and increasing the consumption of anti-inflammatory foods like fruits and vegetables, can help mitigate the chronic inflammatory state observed in both conditions [10,44,47].

Notably, patients with psoriasis and periodontitis are at an increased risk of developing comorbid conditions such as cardiovascular diseases, diabetes, and respiratory conditions. Regular monitoring and management of these comorbidities are crucial. Implementing preventive measures and routine health screenings can help identify and address these risks early, improving patient outcomes and quality of life [6,8].

Educating patients about the interrelated nature of their conditions and the importance of maintaining both oral and skin health can empower them to take an active role in their treatment. Providing resources and support for managing stress, improving diet, and maintaining good oral hygiene can significantly impact disease management [75,76]. Patient empowerment through education can lead to better adherence to treatment protocols and improved overall health [44].

**Figure 3:**
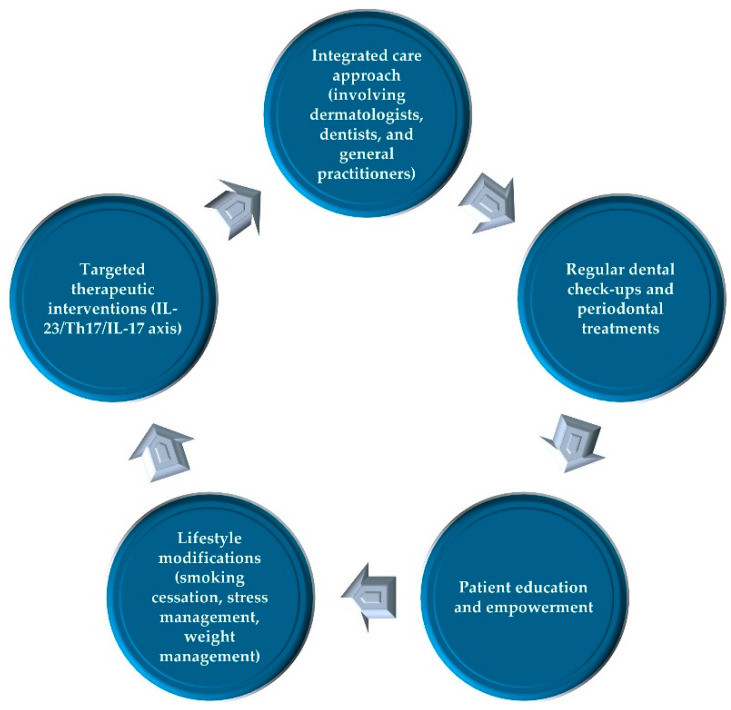
Clinical implications and recommendations for the comorbidity of psoriasis and periodontitis.

In summary, the relationship between psoriasis and periodontitis highlights the importance of an integrated approach to patient care. By addressing both conditions concurrently through comprehensive management strategies, interdisciplinary collaboration, targeted therapies, lifestyle modifications, and patient education, healthcare providers can significantly improve patient outcomes. Understanding and managing the shared pathways and risk factors of these chronic inflammatory diseases can lead to more effective treatments and enhanced quality of life for patients (Figure 3).

## 7. Limits, Strengths, and Future Perspectives

This narrative review provides a comprehensive synthesis of the literature on the complex relationship between psoriasis and periodontitis. However, narrative reviews have certain limitations. They often lack the systematic approach of systematic reviews, and can introduce selection bias due to the subjective selection of included studies. This review may not exhaustively cover all relevant studies, particularly those that do not align with the constructed narrative. Additionally, narrative reviews do not use statistical methods to combine study results, limiting the ability to draw definitive conclusions about the evidence’s strength and consistency.

Despite these limitations, this narrative review has several strengths. It integrates findings from a diverse range of studies, offering a holistic understanding of the multifactorial nature of these diseases, encompassing genetic, immunological, microbial, and environmental factors. It also highlights the importance of an integrated care approach and emphasizes the clinical implications of managing these interrelated conditions. By synthesizing current knowledge, this review serves as a valuable resource for clinicians and researchers, guiding future investigations and informing clinical practice.

To strengthen claims of association and causality, future research should focus on studies that include both psoriasis and periodontitis patients in the same cohorts to ensure a more direct examination of the interplay between these chronic inflammatory diseases. Until then, the emphasis on common disease progression and risk factors remains speculative and requires cautious interpretation in clinical practice.

Future longitudinal studies investigating the effects of periodontal treatment on psoriasis outcomes and vice versa could provide deeper insights into the bidirectional relationship. Understanding how genetic predispositions and environmental factors interact to influence the development and progression of both diseases is crucial for developing more personalized and effective treatment strategies [17]. In particular, developing and testing integrated care models that involve collaboration between dermatologists, dentists, and other healthcare providers can enhance patient outcomes.

Future research should also focus on the temporal relationship between periodontitis and psoriasis; elucidating whether improving periodontal health can positively impact psoriasis outcomes and vice versa; and the molecular and cellular mechanisms underlying the bidirectional relationship between these conditions, paving the way for targeted therapies that address shared inflammatory pathways and genetic and environmental interactions, and lead to more tailored treatment approaches that consider individual patient risk factors, and patient-reported outcomes to understand the impact of these conditions and their treatments on quality of life.

## 8. Conclusions

The interplay between psoriasis and chronic periodontitis involves genetic, immunological, microbial, and environmental factors, as highlighted by epidemiological studies revealing a bidirectional relationship. Shared inflammatory pathways, characterized by dysregulated immune responses and microbial dysbiosis, drive both conditions, leading to tissue damage and chronic inflammation. Genetic insights underscore a potential common genetic basis of both diseases, opening avenues for novel therapeutic strategies targeting shared molecular pathways.

Oral microbiota, particularly periodontal pathogens, may influence psoriasis through microbial dysbiosis, systemic inflammation, immune modulation, and cross-reactivity. Thus, managing periodontal health could reduce systemic inflammation and potentially alleviate psoriasis severity.

Environmental factors such as infections, medication use, psychological stress, and obesity significantly affect the pathogenesis and progression of both diseases. Understanding these influences is essential for developing comprehensive management strategies to reduce disease severity and improve patient outcomes.

Unraveling the pathophysiological mechanisms linking psoriasis and periodontitis is crucial for developing targeted therapies. Interdisciplinary collaboration between dermatologists and periodontists is vital for optimizing patient management. Future research should prioritize exploring the molecular intricacies of this association and innovative treatments to address the complex pathophysiology of both conditions. Recognizing the systemic health factors in managing dermatologic and periodontal conditions can enhance the quality of life for patients with these chronic inflammatory diseases.

## Figures and Tables

**Table 1:** Interleukin-17 (IL-17) in psoriasis and periodontitis pathogenesis.

Interleukin-17 (IL-17)	Psoriasis	Periodontitis
**Cytokine Production and Immune Activation**	IL-17 is primarily produced by Th17 cells, activated by cytokines such as IL-23, promoting differentiation and expansion. Th17 cells secrete IL-17 along with IL-22 and IL-21, recruiting and activating neutrophils and other immune cells at the inflammation site [54,59,60].	IL-17 is produced by Th17 cells in response to bacterial antigens. Th17 cell differentiation and expansion are driven by cytokines such as IL-23, IL-6, and TGF-β. Th17 cells secrete IL-17, inducing pro-inflammatory cytokines, chemokines, and MMPs in various cell types [9,54].
**Keratinocyte Activation and Skin Inflammation**	IL-17 acts on keratinocytes, inducing antimicrobial peptides, pro-inflammatory cytokines (e.g., TNF-α, IL-1β, IL-6), and chemokines (e.g., CXCL1, CXCL2, CXCL8), leading to keratinocyte hyperproliferation and impaired differentiation, resulting in psoriatic plaques [58,61].	IL-17 induces pro-inflammatory cytokines, chemokines, and MMPs in epithelial cells, fibroblasts, and osteoblasts, contributing to the inflammatory environment and tissue damage in periodontitis [9].
**Neutrophil Recruitment**	IL-17 induces the expression of chemokines that attract neutrophils to the inflammation site, resulting in Munro’s microabscesses, a hallmark of psoriasis [9,58].	IL-17 is a potent inducer of neutrophil recruitment, which forms a major component of the inflammatory infiltrate in periodontitis. Neutrophils help control infections but can cause tissue damage through ROS and proteolytic enzymes [9,47].
**Interaction with Other Cytokines**	IL-17 works synergistically with cytokines like TNF-α and IL-22, enhancing inflammatory responses and creating a chronic inflammation environment. The IL-17/TNF-α axis is crucial in psoriasis, where they amplify each other’s effects [9,58,60].	IL-17 synergizes with TNF-α and IL-1β, amplifying inflammatory responses in periodontal tissues, creating a feedback loop that perpetuates chronic inflammation and tissue damage [9,17].
**Bone and Joint Involvement**	IL-17 is implicated in psoriatic arthritis by promoting osteoclastogenesis, leading to bone erosion and joint damage. IL-17-induced inflammation in the joints mirrors its effects in the skin, contributing to synovial hyperplasia, cartilage damage, and bone destruction [4,58,60].	IL-17 stimulates osteoclastogenesis by upregulating RANKL and downregulating OPG, leading to alveolar bone destruction in periodontitis. This process results in tooth loss if left untreated [47,54].
**Therapeutic Target**	IL-17 inhibitors, such as secukinumab and ixekizumab, are highly effective in reducing psoriasis severity by blocking IL-17 activity, demonstrating significant clinical efficacy in clearing psoriatic plaques and improving patients’ quality of life [62,63].	IL-17 is a potential therapeutic target in periodontitis. Inhibitors of IL-17 or its pathways could reduce inflammation and prevent tissue destruction in periodontal disease. Clinical studies targeting IL-17 in psoriasis have shown promise, suggesting that similar approaches could be effective in periodontitis [62,63].

**Abbreviations:** Interleukin-17, IL-17; T-helper 17, Th17; Interleukin-23, IL-23; Interleukin-22, IL-22; Interleukin-21, IL-21; Interleukin-6, IL-6; Transforming Growth Factor-beta, TGF-β; Matrix Metalloproteinases, MMPs; Tumor Necrosis Factor-alpha, TNF-α; Interleukin-1 beta, IL-1β; Chemokine (C-X-C motif) Ligand 1, CXCL1; Chemokine (C-X-C motif) Ligand 2, CXCL2; Chemokine (C-X-C motif) Ligand 8, CXCL8; Reactive Oxygen Species, ROS; Receptor Activator of Nuclear Factor kappa-B Ligand, RANKL; Osteoprotegerin, OPG.
